# Para Além do Humor: O Riso como Estímulo Cardiovascular

**DOI:** 10.36660/abc.20250886

**Published:** 2026-04-30

**Authors:** Ricardo Stein, Cleidiane da Silva Andrade, Filipe Ferrari

**Affiliations:** 1 Universidade Federal do Rio Grande do Sul Faculdade de Medicina Porto Alegre RS Brasil Programa de Pós-Graduação em Cardiologia e Ciências Cardiovasculares – Faculdade de Medicina – Universidade Federal do Rio Grande do Sul, Porto Alegre, RS – Brasil; 2 Hospital de Clínicas de Porto Alegre Grupo de Pesquisa em Cardiologia do Exercício (CardioEx) Porto Alegre RS Brasil Grupo de Pesquisa em Cardiologia do Exercício (CardioEx) – Hospital de Clínicas de Porto Alegre, Porto Alegre, RS – Brasil; 3 Universidade Federal do Rio Grande do Sul Departamento de Clínica Médica Porto Alegre RS Brasil Departamento de Clínica Médica – Universidade Federal do Rio Grande do Sul, Porto Alegre, RS – Brasil

**Keywords:** Terapia do Riso, Reabilitação Cardíaca, Doenças Cardiovasculares

## Por que o riso é importante para o sistema cardiovascular?

A doença cardiovascular (DCV) continua sendo a principal causa de mortalidade em todo o mundo,^1^ apesar dos avanços substanciais nas terapias farmacológicas e intervencionistas. Nesse contexto, estratégias não farmacológicas, de baixo custo, escaláveis e acessíveis, têm despertado crescente interesse. A terapia do riso (TR), tradicionalmente associada ao bem-estar psicológico, surgiu como um estímulo fisiológico adjunto capaz de provocar respostas cardiovasculares mensuráveis.^2-5^

**Figura 1:**
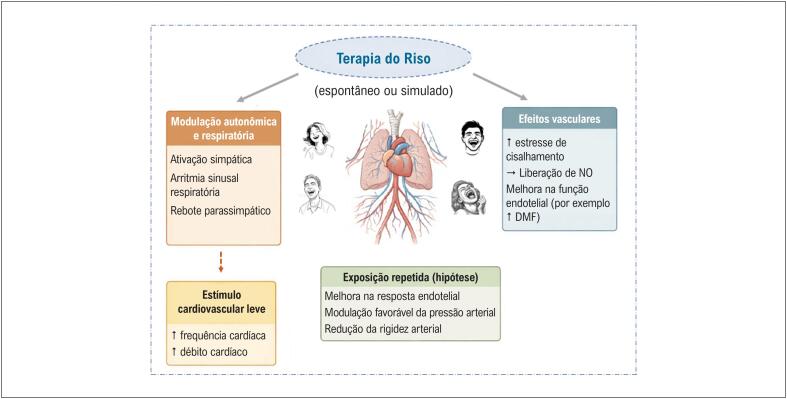
Mecanismos propostos que ligam a terapia do riso aos benefícios cardiovasculares. Estrutura conceitual que ilustra a terapia do riso (espontâneo ou simulado) como um potencial estímulo cardiovascular de baixa intensidade. O riso pode induzir modulação autonômica e respiratória aguda, caracterizada por ativação simpática transitória, arritmia sinusal respiratória e subsequente rebote parassimpático. Essas respostas podem levar a aumentos leves na frequência cardíaca e no débito cardíaco, juntamente com estresse de cisalhamento vascular e liberação de óxido nítrico, contribuindo para melhorias de curto prazo na função endotelial. A exposição repetida é hipotetizada como capaz de aprimorar a responsividade endotelial, modular favoravelmente a pressão arterial e reduzir a rigidez arterial. DMF: dilatação mediada por fluxo.

Dados observacionais sugerem que indivíduos com doença arterial coronariana riem com menor frequência e apresentam níveis mais elevados de raiva e hostilidade — traços psicológicos associados a desfechos cardiovasculares adversos.^6^ Além disso, menor frequência diária de riso tem sido associada ao aumento da incidência e da mortalidade por DCV.^7,8^ Embora essas associações não estabeleçam causalidade, levantam uma questão fisiológica importante: o riso em si pode funcionar como um estímulo cardiovascular relevante? As vias fisiológicas hipotéticas pelas quais a TR pode influenciar a regulação autonômica, a hemodinâmica e a função vascular estão resumidas na Figura 1.

Argumentamos que o riso não deve ser visto apenas como um fenômeno emocional ou comportamental. Em vez disso, as evidências disponíveis sustentam o conceito de que o riso atua como um estressor cardiovascular de baixa intensidade, capaz de modular o equilíbrio autonômico, a função vascular e a hemodinâmica por meio de mecanismos que se assemelham parcialmente aos observados durante a atividade física leve.^9^

## Escopo e perspectivas da literatura

Este ponto de vista baseia-se em uma avaliação narrativa da literatura disponível que examina as respostas cardiovasculares relacionadas ao riso. Em vez de buscar uma cobertura exaustiva, focamos em estudos experimentais representativos e em estudos clínicos exploratórios que destacam conceitos fisiológicos centrais e achados recorrentes em diferentes contextos. A seleção dos estudos foi guiada pela relevância para os mecanismos cardiovasculares e pela contribuição de cada um para o arcabouço conceitual discutido neste trabalho.

Em conformidade com o formato "Ponto de Vista", não foi aplicada uma estratégia sistemática formal de busca nem avaliação de risco de viés, e a possibilidade de viés de seleção não pode ser excluída. Assim, as evidências citadas devem ser interpretadas como ilustrativas e geradoras de hipóteses, em vez de definitivas ou exaustivas.

## O riso como estressor cardiovascular de baixa intensidade

O riso envolve a ativação coordenada dos músculos faciais, respiratórios, torácicos, e abdominais, resultando em ciclos respiratórios rítmicos e aumentos transitórios da pressão intratorácica. Esse envolvimento muscular e respiratório está associado à estimulação cardiovascular aguda, refletida de forma mais consistente por aumentos transitórios da frequência cardíaca (FC) e do débito cardíaco.^10^

Estudos experimentais demonstraram que episódios de riso espontâneo durante a visualização de comédias podem aumentar a FC em aproximadamente 20%, com respostas proporcionais à duração e à intensidade do riso.^9^ Essas alterações são comparáveis às observadas durante atividade física leve a moderada ^9^ e parecem ser influenciadas pelo contexto social, já que o riso tende a ser mais frequente e intenso quando os indivíduos são expostos a estímulos humorísticos em ambientes familiares ou socialmente envolventes.^11^

O riso simulado, caracterizado por risadas intencionais e repetitivas sem gatilhos emocionais, frequentemente induz respostas cardiovasculares mais pronunciadas.^12^ Isso provavelmente reflete maior frequência respiratória e maior envolvimento muscular, reforçando o conceito de que o ato fisiológico de rir, mais do que apenas a percepção do humor, é o responsável por esses efeitos.

Do ponto de vista autonômico, o riso representa um estado de alta excitação, caracterizado por ativação simpática e retirada parassimpática, seguido por uma fase de recuperação marcada por aumento da atividade parassimpática.^13^ A arritmia sinusal respiratória desempenha papel central nesse processo, acoplando os ciclos respiratórios à variabilidade da FC e potencialmente otimizando as trocas gasosas. Esse padrão autonômico se assemelha estreitamente ao observado durante o exercício aeróbico leve.^14-17^

Em períodos mais longos, a exposição repetida ao riso tem sido associada a reduções da FC de repouso e dos níveis de hormônios do estresse, sugerindo uma mudança em direção a um equilíbrio autonômico aprimorado. Coletivamente, essas observações sustentam a hipótese de que o riso pode exercer tanto efeitos cardiovasculares agudos de estimulação quanto efeitos regulatórios de longo prazo.

## Efeitos vasculares e hemodinâmicos: Para além da emoção

Além da modulação autonômica, a TR tem sido associada a efeitos consistentes, embora de curta duração, sobre a função vascular. Ensaios cruzados utilizando dilatação mediada por fluxo demonstraram que a exposição a filmes de comédia pode melhorar agudamente a vasodilatação dependente do endotélio,^18,19^ com magnitudes comparáveis às observadas após exercício aeróbico.^20,21^

Essas respostas vasculares são consideradas mediadas pelo aumento do estresse de cisalhamento decorrente das elevações induzidas pelo riso no débito cardíaco e no fluxo sanguíneo, potencialmente estimulando a liberação de óxido nítrico. Mecanismos adicionais podem incluir reduções em mediadores neuroendócrinos vasoconstritores e ativação de vias relacionadas às endorfinas, que poderiam aprimorar ainda mais a função endotelial.

O riso também tem sido associado a reduções de curto prazo na rigidez arterial, avaliadas pela velocidade da onda de pulso, e a mudanças favoráveis em biomarcadores de lesão endotelial.^22^ Importante destacar que esses efeitos parecem se dissipar em poucas horas ou dias, particularmente em indivíduos jovens e saudáveis, ressaltando seu caráter predominantemente agudo.

No entanto, o fato de ser transitório não implica que seja trivial. As respostas vasculares agudas ao exercício também são de curta duração, a menos que sejam reforçadas por treinamento regular. Esse paralelo levanta uma questão crítica, ainda pouco explorada: a exposição repetida ao riso poderia induzir adaptações vasculares cumulativas ao longo do tempo? Embora os dados de longo prazo permaneçam limitados, evidências emergentes sugerem que essa hipótese merece investigação sistemática, particularmente em populações com disfunção endotelial.

## Demanda metabólica e capacidade funcional

Apesar de seus efeitos cardiovasculares, a TR parece impor uma demanda metabólica relativamente baixa. Estudos que avaliaram o consumo de oxigênio (VO_2_) durante o riso relataram aumentos mínimos ou inexistentes, com gasto energético comparável ao de atividades leves do cotidiano.^9,23^ Essa aparente dissociação entre a estimulação hemodinâmica central e a extração periférica de oxigênio é consistente com a noção de que o riso pode aumentar o débito cardíaco sem elevar substancialmente a carga metabólica.

Essa característica pode ser particularmente relevante em populações clínicas com tolerância limitada ao exercício. Dados preliminares sugerem que a exposição repetida ao riso pode induzir respostas fisiológicas modestas relacionadas à regulação cardiovascular em populações clínicas selecionadas;^19,24^ no entanto, essas observações derivam de pequenos estudos exploratórios e devem ser interpretadas com cautela.

Do ponto de vista fisiológico, a TR pode, portanto, ocupar um nicho distinto, fornecendo estimulação cardiovascular suficiente para desencadear respostas adaptativas, ao mesmo tempo em que permanece acessível a indivíduos incapazes de participar de programas estruturados de exercício.

## O que o riso não substitui: diferenças fundamentais em relação ao exercício físico

Embora os paralelos entre as respostas cardiovasculares induzidas pelo riso e a atividade física leve sejam úteis do ponto de vista fisiológico, é essencial enfatizar que intervenções baseadas no riso não replicam os princípios centrais do treinamento físico estruturado. O riso não envolve sobrecarga progressiva, produz um estímulo metabólico substancialmente menor e menos duradouro, e não promove adaptações musculoesqueléticas, como ganhos de força, resistência ou capacidade oxidativa do músculo esquelético.

Além disso, o treinamento físico induz adaptações centrais e periféricas bem estabelecidas, incluindo melhorias na função mitocondrial, na capilarização muscular e na sensibilidade à insulina – efeitos que não foram demonstrados com a TR. Assim, qualquer comparação com o exercício físico deve ser interpretada estritamente como uma analogia fisiológica, e não como equivalência funcional ou terapêutica. O riso deve, portanto, ser visto, no máximo, como um estímulo fisiológico complementar, e não como substituto da atividade física estruturada ou da reabilitação baseada em exercício.

## Significado clínico: Quem pode se beneficiar?

Do ponto de vista conceitual, atualmente não há evidências de que a TR melhore desfechos cardiovasculares duros, como eventos cardiovasculares ou mortalidade, e os dados disponíveis devem ser interpretados como sinais fisiológicos exploratórios. A maior parte das evidências fisiológicas deriva de estudos conduzidos em indivíduos saudáveis ou em pequenas amostras exploratórias, e a extrapolação para pacientes com DCV estabelecida, idosos ou indivíduos com limitações funcionais permanece, portanto, hipotética.

Nesse contexto, a TR tem sido discutida conceitualmente como um estímulo adjunto potencial para o engajamento cardiovascular em populações com tolerância limitada ao exercício. Contudo, tais considerações são teóricas e não devem ser interpretadas como recomendações práticas, especialmente na ausência de ensaios clínicos dedicados que avaliem segurança, viabilidade e eficácia em populações de alto risco.

Importante destacar que a TR deve ser vista como um estímulo fisiológico, e não como uma intervenção terapêutica, e qualquer sobreposição com vias relacionadas ao exercício não deve ser interpretada como equivalência funcional.

## O que ainda não sabemos

Apesar dos achados encorajadores, permanecem lacunas importantes no conhecimento. Protocolos ideais em relação à frequência, duração e modo de indução do riso ainda não foram estabelecidos. Os desfechos cardiovasculares de longo prazo não foram adequadamente estudados, e os dados em populações de alto risco ou com múltiplas comorbidades continuam limitados.

Além disso, as relações dose-resposta e as possíveis interações com o treinamento físico convencional ainda precisam ser exploradas. Abordar essas lacunas será essencial antes que a TR possa ser avaliada em programas estruturados de prevenção ou reabilitação cardiovascular.

## Distinção conceitual entre o riso como estímulo fisiológico e como intervenção terapêutica

É importante distinguir claramente o riso como fenômeno fisiológico das intervenções baseadas no riso propostas como estratégias terapêuticas. O riso espontâneo representa uma resposta emocional e comportamental que ocorre naturalmente, tipicamente inserida na interação social, enquanto o riso, simulado envolve padrões motores voluntários de riso sem um gatilho humorístico externo. Programas estruturados de TR, por sua vez, combinam elementos de riso simulado, técnicas de respiração e dinâmicas de grupo dentro de um protocolo pré-definido, sendo frequentemente enquadrados como intervenções comportamentais.

A maior parte das evidências fisiológicas disponíveis refere-se às respostas agudas a episódios de riso, incluindo modulação transitória da atividade autonômica, da função vascular e de parâmetros hemodinâmicos. Em contraste, a noção da TR como intervenção terapêutica sustentada permanece em grande parte hipotética, já que dados longitudinais demonstrando adaptações fisiológicas duradouras ou benefício clínico são escassos. Assim, os achados existentes devem ser interpretados principalmente como evidência de um estímulo fisiológico, e não como prova de eficácia terapêutica.

## Considerações metodológicas e limitações

Apesar da plausibilidade fisiológica e da coerência interna dos achados disponíveis, o corpo atual de evidências que sustenta os efeitos cardiovasculares induzidos pela TR apresenta importantes limitações metodológicas que exigem interpretação cautelosa. A maioria dos estudos é caracterizada por tamanhos amostrais reduzidos, períodos de intervenção curtos e poder estatístico limitado, aumentando a suscetibilidade a erros aleatórios e à superestimação dos efeitos.

O cegamento é, por natureza, desafiador em TR, o que aumenta a possibilidade de efeitos de expectativa e de Hawthorne, particularmente em ambientes de interação social. Além disso, há substancial heterogeneidade nos protocolos de indução do riso, variando desde riso espontâneo ou simulado até programas estruturados de TR, com marcada variabilidade na duração, frequência, intensidade e contexto social das sessões.

A ausência de grupos-controle ativos em diversos estudos limita ainda mais a inferência causal, enquanto persistem dificuldades em padronizar objetivamente a intensidade do riso e em quantificar relações dose-resposta. Por fim, não se pode excluir a possibilidade de viés de publicação, dado o predomínio de pequenos estudos exploratórios e pilotos que relatam respostas fisiológicas favoráveis. Coletivamente, essas limitações reforçam que as evidências atuais devem ser vistas como geradoras de hipóteses, e não como confirmatórias, e que a extrapolação para desfechos clínicos ou eficácia terapêutica requer ensaios dedicados, bem controlados.

## Conclusão

Diante das limitações metodológicas da literatura existente, as evidências disponíveis são consistentes com a visão de que a TR pode funcionar como um estímulo cardiovascular de baixa intensidade, capaz de modular a atividade autonômica, a função vascular e parâmetros hemodinâmicos sem aumentar substancialmente a demanda metabólica. Embora os dados atuais ainda sejam limitados, achados emergentes sugerem que a exposição regular ao riso pode oferecer benefícios fisiologicamente plausíveis, particularmente para indivíduos com capacidade reduzida para o exercício. Argumentamos que o riso merece consideração como uma abordagem conceitual complementar no âmbito do discurso e da pesquisa em prevenção cardiovascular, e que justifica investigação rigorosa em futuros ensaios clínicos.

## Data Availability

Os conteúdos subjacentes ao texto da pesquisa estão contidos no manuscrito.
