# Supplementary material for: Para Além do Humor: O Riso como Estímulo Cardiovascular
Source: Arq Bras Cardiol. 2026 Apr 30;123(3):e20250886. [Article in Portuguese] doi: 10.36660/abc.20250886 (PMC13155791; doi:10.36660/abc.20250886)
Supplement: Translation

# Translation: Beyond Humor: Laughter as a Cardiovascular Stimulus

**DOI:** 10.36660/abc.20250886i

**Authors:** Ricardo Stein, Cleidiane da Silva Andrade, Filipe Ferrari

**Affiliations:** 1 Porto Alegre RS Brazil Programa de Pós-Graduação em Cardiologia e Ciências Cardiovasculares – Faculdade de Medicina – Universidade Federal do Rio Grande do Sul, Porto Alegre, RS – Brazil; 2 Porto Alegre RS Brazil Grupo de Pesquisa em Cardiologia do Exercício (CardioEx) – Hospital de Clínicas de Porto Alegre, Porto Alegre, RS – Brazil; 3 Porto Alegre RS Brazil Departamento de Clínica Médica – Universidade Federal do Rio Grande do Sul, Porto Alegre, RS – Brazil

**Keywords:** Laughter Therapy, Cardiac Rehabilitation, Cardiovascular Diseases

## Why Laughter Matters to the Cardiovascular System

Cardiovascular disease (CVD) remains the leading cause of mortality worldwide,^1^ despite substantial advances in pharmacological and interventional therapies. In this context, non-pharmacological strategies that are low-cost, scalable, and accessible have attracted growing interest. Laughter therapy (LT), traditionally associated with psychological well-being, has emerged as a potential adjunctive physiological stimulus capable of eliciting measurable cardiovascular responses.^2-5^

Observational data suggest that individuals with coronary artery disease laugh less frequently and exhibit higher levels of anger and hostility – psychological traits associated with adverse cardiovascular outcomes.^6^ Moreover, lower daily laughter frequency has been linked to increased CVD incidence and mortality.^7,8^ While these associations do not establish causality, they raise an important physiological question: can laughter itself act as a meaningful cardiovascular stimulus? The hypothesized physiological pathways through which LT may influence autonomic regulation, hemodynamics, and vascular function are summarized in Figure 1.

**Figure 1:** Proposed mechanisms linking laughter therapy with cardiovascular benefits. Conceptual framework illustrating laughter therapy (spontaneous or simulated) as a potential low-intensity cardiovascular stimulus. Laughter may induce acute autonomic and respiratory modulation, characterized by transient sympathetic activation, respiratory sinus arrhythmia, and subsequent parasympathetic rebound. These responses may lead to mild increases in heart rate and cardiac output, along with vascular shear stress and nitric oxide release, contributing to short-term improvements in endothelial function. Repeated exposure is hypothesized to enhance endothelial responsiveness, favorably modulate blood pressure, and reduce arterial stiffness. FMD: flow-mediated dilation.

We argue that laughter should not be viewed solely as an emotional or behavioral phenomenon. Instead, available evidence supports the concept of laughter as a low-intensity cardiovascular stressor, capable of modulating autonomic balance, vascular function, and hemodynamics through mechanisms that partially resemble those observed during light physical activity.^9^

## Literature scope and perspective

This Viewpoint is based on a narrative appraisal of the available literature examining laughter-related cardiovascular responses. Rather than aiming for exhaustive coverage, we focused on representative experimental and exploratory clinical studies that illustrate key physiological concepts and recurring findings across different settings. Study selection was guided by relevance to cardiovascular mechanisms and contribution to the conceptual framework discussed herein.

Consistent with the Viewpoint format, no formal systematic search strategy or risk of bias assessment was applied, and the possibility of selection bias cannot be excluded. Accordingly, the cited evidence should be interpreted as illustrative and hypothesis-generating, rather than definitive or comprehensive.

## Laughter as a low-intensity cardiovascular stressor

Laughter involves coordinated activation of facial, respiratory, thoracic, and abdominal muscles, resulting in rhythmic respiratory cycles and transient increases in intrathoracic pressure. This muscular and respiratory engagement is associated with acute cardiovascular stimulation, most consistently reflected by transient increases in heart rate (HR) and cardiac output.^10^

Experimental studies have shown that episodes of spontaneous laughter during comedy viewing can increase HR by approximately 20%, with responses proportional to laughter duration and intensity.^9^ These changes are comparable to those observed during mild-to-moderate physical activity^9^ and appear to be influenced by social context, as laughter tends to be more frequent and intense when individuals are exposed to humorous stimuli in familiar or socially engaging environments.^11^

Simulated laughter, characterized by intentional and repetitive laughter without emotional triggers, often induces more pronounced cardiovascular responses.^12^ This likely reflects greater respiratory frequency and muscular engagement, reinforcing the concept that the physiological act of laughing, rather than humor perception alone, drives these effects.

From an autonomic standpoint, laughter represents a high-arousal state characterized by sympathetic activation and parasympathetic withdrawal, followed by a recovery phase marked by enhanced parasympathetic activity.^13^ Respiratory sinus arrhythmia plays a central role in this process, coupling respiratory cycles with HR variability and potentially optimizing gas exchange. This autonomic pattern closely resembles that observed during light aerobic exercise.^14-17^

Over longer periods, repeated laughter exposure has been associated with reductions in resting HR and stress hormone levels, suggesting a shift toward improved autonomic balance. Collectively, these observations support the hypothesis that laughter may exert both acute stimulatory and long-term regulatory cardiovascular effects.

## Vascular and hemodynamic effects: Beyond emotion

Beyond autonomic modulation, LT has been associated with consistent, albeit short-lived, effects on vascular function. Crossover trials using flow-mediated dilation have shown that exposure to comedy films can acutely improve endothelium-dependent vasodilation,^18,19^ with magnitudes comparable to those observed after aerobic exercise.^20,21^

These vascular responses are thought to be mediated by increased shear stress resulting from laughter-induced elevations in cardiac output and blood flow, potentially stimulating nitric oxide release. Additional mechanisms may include reductions in vasoconstrictive neuroendocrine mediators and activation of endorphin-related pathways that could further enhance endothelial function.

Laughter has also been associated with short-term reductions in arterial stiffness, as assessed by pulse wave velocity, and favorable changes in biomarkers of endothelial injury.^22^ Importantly, these effects appear to dissipate within hours to days, particularly in young, healthy individuals, underscoring their predominantly acute nature.

However, transient does not mean trivial. Acute vascular responses to exercise are similarly short-lived unless reinforced by regular training. This parallel raises a critical yet underexplored question: could repeated laughter exposure induce cumulative vascular adaptations over time? While long-term data remain limited, emerging evidence suggests that this hypothesis warrants systematic investigation, particularly in populations with endothelial dysfunction.

## Metabolic demand and functional capacity

Despite its cardiovascular effects, LT appears to impose a relatively low metabolic demand. Studies assessing oxygen consumption (VO_2_) during laughter have reported minimal or no increases, with energy expenditure comparable to that of light daily activities.^9,23^ This apparent dissociation between central hemodynamic stimulation and peripheral oxygen extraction is consistent with the notion that laughter may increase cardiac output without substantially increasing metabolic load.

This characteristic may be particularly relevant in clinical populations with limited exercise tolerance. Preliminary data suggest that repeated laughter-based exposure may induce modest physiological responses related to cardiovascular regulation in selected clinical populations;^19,24^ however, these observations derive from small exploratory studies and must be interpreted with caution.

From a physiological perspective, LT may therefore occupy a distinctive niche, providing cardiovascular stimulation sufficient to elicit adaptive responses while remaining accessible to individuals unable to engage in structured exercise programs.

## What laughter does not replace: Key differences from physical exercise

Although parallels between laughter-induced cardiovascular responses and light physical activity are useful from a physiological perspective, it is essential to emphasize that laughter-based interventions do not replicate the core training principles of structured exercise. Laughter lacks progressive overload, produces a substantially lower and less sustained metabolic stimulus, and does not promote musculoskeletal adaptations such as improvements in strength, endurance, or skeletal muscle oxidative capacity.

Moreover, exercise training induces well-established central and peripheral adaptations, including improvements in mitochondrial function, muscle capillarization and insulin sensitivity, which have not been demonstrated with LT. Accordingly, any comparison with physical exercise should be interpreted strictly as a physiological analogy rather than a functional or therapeutic equivalence. Laughter should therefore be viewed, at most, as a complementary physiological stimulus, not as a substitute for structured physical activity or exercise-based rehabilitation.

## Clinical meaning: Who might benefit?

From a conceptual standpoint, there is currently no evidence that LT improves hard cardiovascular outcomes, such as cardiovascular events or mortality, and available data should be interpreted as exploratory physiological signals. Most physiological evidence derives from studies conducted in healthy individuals or small exploratory samples, and extrapolation to patients with established CVD, older adults, or individuals with functional limitations therefore remains hypothetical.

In this context, LT has been conceptually discussed as potential adjunctive stimuli for cardiovascular engagement in populations with limited exercise tolerance. However, such considerations are theoretical and should not be interpreted as practice recommendations, particularly in the absence of dedicated clinical trials evaluating safety, feasibility, and efficacy in high-risk populations.

Importantly, LT should be viewed as a physiological stimulus rather than as a therapeutic intervention, and any overlap with exercise-related pathways should not be interpreted as functional equivalence.

## What we still do not know

Despite encouraging findings, important knowledge gaps remain. Optimal protocols regarding the frequency, duration, and mode of laughter induction have not been established. Long-term cardiovascular outcomes have not been adequately studied, and data in high-risk or multimorbid populations remain limited.

Moreover, dose-response relationships and potential interactions with conventional exercise training have yet to be explored. Addressing these gaps will be essential before LT can be considered for evaluation in structured cardiovascular prevention or rehabilitation programs.

## Conceptual distinction between laughter as a physiological stimulus and as a therapeutic intervention

It is important to clearly distinguish laughter as a physiological phenomenon from laughter-based interventions proposed as therapeutic strategies. Spontaneous laughter represents a naturally occurring emotional and behavioral response, typically embedded in social interaction, whereas simulated laughter involves voluntary motor patterns of laughter without an external humorous trigger. Structured LT programs, in turn, combine elements of simulated laughter, breathing techniques, and group dynamics within a predefined protocol and are often framed as behavioral interventions.

Most of the available physiological evidence relates to acute responses to laughter episodes, including transient modulation of autonomic activity, vascular function, and hemodynamic parameters. In contrast, the notion of LT as a sustained therapeutic intervention remains largely hypothetical, as longitudinal data demonstrating durable physiological adaptations or clinical benefit are scarce. Accordingly, existing findings should be interpreted primarily as evidence of a physiological stimulus rather than proof of therapeutic efficacy.

## Methodological considerations and limitations

Despite the physiological plausibility and internal coherence of the available findings, the current body of evidence supporting LT-induced cardiovascular effects is subject to important methodological limitations that warrant cautious interpretation. Most studies are characterized by small sample sizes, short intervention periods, and limited statistical power, increasing susceptibility to random error and overestimation of effects.

Blinding is inherently challenging in LT, raising the possibility of expectation and Hawthorne effects, particularly in socially interactive settings. In addition, there is substantial heterogeneity in laughter-induction protocols, ranging from spontaneous or simulated laughter to structured LT programs, with marked variability in session duration, frequency, intensity, and social context.

The absence of active control groups in several studies further limits causal inference, while difficulties in objectively standardizing laughter intensity and quantifying dose-response relationships remain unresolved. Finally, potential publication bias cannot be excluded, given the predominance of small exploratory and pilot studies reporting favorable physiological responses. Collectively, these limitations underscore that current evidence should be viewed as hypothesis-generating rather than confirmatory, and that extrapolation to clinical outcomes or therapeutic effectiveness requires dedicated, well-controlled trials.

## Conclusion

In light of the methodological limitations of the existing literature, available evidence is consistent with the view that LT may function as a low-intensity cardiovascular stimulus capable of modulating autonomic activity, vascular function, and hemodynamics without substantially increasing metabolic demand. While current data remain limited, emerging findings suggest that regular laughter exposure may offer physiologically plausible benefits, particularly for individuals with restricted exercise capacity. We argue that laughter merits consideration as a conceptual complementary approach within cardiovascular prevention discourse and research and warrants rigorous investigation in future clinical trials.

## Data Availability

The underlying content of the research text is contained within the manuscript.
